# First-Principles Study of the Superconductivity of Ti_3_VH_12_ and TiV_3_H_12_ Under 200 GPa

**DOI:** 10.3390/ma19153171

**Published:** 2026-07-24

**Authors:** Jing Luo, Qun Wei, Meiguang Zhang

**Affiliations:** 1 School of Physics, Xidian University, Xi’an 710071, China; luoj0225@163.com; 2 College of Physics and Optoelectronic Technology, Baoji University of Arts and Sciences, Baoji 721016, China

**Keywords:** superconductivity, electron-phonon coupling, first-principles calculations, hydride

## Abstract

Hydrogen-rich compounds under high pressure are promising for high-temperature superconductivity, but many high-$T_{c}$ hydrides rely on rare-earth or alkaline-earth elements and remain difficult to tune chemically. Transition-metal hydrides offer an alternative platform because partially filled *d* states can modify the electronic density of states, metal–hydrogen hybridization, and electron–phonon coupling. Here, ${\mathrm{VH}}_{3}$ is used as a parent high-pressure transition-metal hydride framework, and Ti substitution is introduced as a chemically compatible way to tune the *d*-derived states near the Fermi level. Two ternary hydrides, ${\mathrm{Ti}}_{3}{\mathrm{VH}}_{12}$ and ${\mathrm{TiV}}_{3}H_{12}$, are therefore constructed from the ${\mathrm{VH}}_{3}$ lattice and investigated by first-principles calculations at 200 GPa. Both compounds are thermodynamically and dynamically stable under this pressure condition, as indicated by formation energies, the Ti–V–H convex hull, and phonon spectra. Within the same ultrasoft-pseudopotential computational framework, ${\mathrm{Ti}}_{3}{\mathrm{VH}}_{12}$ and ${\mathrm{TiV}}_{3}H_{12}$ yield Allen–Dynes $T_{c}$ values of 42.1 K and 36.8 K, respectively, higher than the corresponding ${\mathrm{VH}}_{3}$ value. A norm-conserving cross-check for ${\mathrm{VH}}_{3}$ gives a different absolute value, indicating that the $T_{c}$ estimates are method-dependent. Electronic structure analysis indicates that Ti incorporation shifts pronounced van Hove singularities close to the Fermi level, enhances the density of states, and changes the Fermi surface topology. These results suggest that Ti–V–H hydrides are a useful model system for examining how transition-metal substitution can couple structural stability with electronic tuning in compressed hydride superconductors.

## 1. Introduction

In recent years, significant progress in high-temperature superconductivity has been driven by the discovery of superconductivity in hydrogen-rich compounds under high pressure. Notably, a superconducting transition temperature ($T_{c}$) exceeding 200 K was experimentally observed in compressed $H_{3}$S, challenging the conventional limits of phonon-mediated superconductivity [1]. According to BCS theory [2], a high $T_{c}$ requires high-frequency phonons, strong electron–phonon coupling, and a large density of states at the Fermi level. Metallic hydrogen is thus predicted to be an ideal high-$T_{c}$ superconductor due to its extremely high phonon frequencies [3,4]. Therefore, the metallization of hydrogen has been regarded as one of the central focuses in current condensed matter physics research [5,6,7,8]. However, its realization requires ultrahigh pressures (>425 GPa) [9]. To overcome this limitation, Ashcroft proposed that hydrogen-rich compounds could mimic metallic hydrogen via chemical precompression, enabling high-$T_{c}$ superconductivity at more accessible pressures [10].

A number of high-pressure hydride superconductors have been reported or predicted, including ${\mathrm{LaH}}_{10}$ (170 GPa, 250 K) [11], ${\mathrm{YCaH}}_{12}$ (180 GPa, 230 K) [12], ${\mathrm{CaH}}_{6}$ (150 GPa, 235 K) [13], ${\mathrm{YZrH}}_{18}$ (200 GPa, 156 K) [14], and ${\mathrm{Li}}_{2}{\mathrm{MgH}}_{16}$ (250 GPa, 473 K) [15]. These examples demonstrate the potential of hydrogen-rich lattices, but they also highlight a persistent materials-design bottleneck. Many high-$T_{c}$ hydrides are based on rare-earth or alkaline-earth elements, whose superconducting properties are often governed by pressure-stabilized hydrogen cages and are less easily adjusted through local electronic-structure control [16]. Developing hydride systems in which the metal sublattice can be chemically tuned is therefore important for moving from isolated high-$T_{c}$ candidates toward clearer design principles.

Transition-metal hydrides are attractive in this context because their partially filled *d* orbitals provide an additional handle for tuning electronic states near the Fermi level. According to the Gaspari–Gyorffy framework [17], electron–phonon coupling in metals is closely related to orbital-dependent scattering at the Fermi surface. In transition-metal hydrides, the same *d* states can strengthen metal–hydrogen hybridization and enhance the density of states at the Fermi level, but they may also localize carriers or reduce the effectiveness of hydrogen-derived phonon modes. This competition makes transition-metal hydrides a useful testing ground for identifying when *d*-electron tuning can improve superconductivity rather than simply adding metallic states.

${\mathrm{VH}}_{3}$ was selected as the parent framework because vanadium hydrides are among the transition-metal hydrides predicted to become stable and metallic under compression [18,19]. Unlike a search that begins from an isolated elemental $T_{c}$ value, ${\mathrm{VH}}_{3}$ provides a concrete high-pressure V–H lattice in which metal-centered hydrogen cages, *d*-derived bands, and electron–phonon coupling can be compared before and after substitution. Ti was chosen as a substituent because it is chemically compatible with V in the transition-metal sublattice while having a different *d*-electron count, allowing the electronic structure near the Fermi level to be adjusted without destroying the ${\mathrm{VH}}_{3}$-type hydrogen framework. The resulting Ti–V–H compositions, ${\mathrm{Ti}}_{3}{\mathrm{VH}}_{12}$ and ${\mathrm{TiV}}_{3}H_{12}$, therefore provide a focused model for testing whether transition-metal substitution can simultaneously stabilize hydrogen-rich frameworks and tune van Hove features, Fermi-surface complexity, and electron–phonon coupling. By comparing these ternary hydrides with ${\mathrm{VH}}_{3}$, the present study examines Ti substitution as a design strategy for improving superconductivity in compressed transition-metal hydrides.

## 2. Computational Methods

Structural optimizations and ground-state electronic properties were calculated within the framework of density functional theory (DFT) as implemented in the Vienna Ab initio Simulation Package (VASP) [20]. The projector augmented-wave (PAW) method was used to describe the electron–ion interaction [21], and the exchange–correlation functional was treated within the generalized gradient approximation (GGA) using the Perdew–Burke–Ernzerhof (PBE) formulation [22]. The plane-wave basis set was expanded with a kinetic energy cutoff of 600 eV. Brillouin zone integrations were carried out using Monkhorst–Pack k-point meshes with a resolution of $2\pi \times 0.02\;{Å}^{-1}$ [23]. Structural optimizations were performed with an energy convergence criterion of $1\times 10^{-5}$ eV/atom. Phonon calculations were performed within the framework of density functional perturbation theory (DFPT) using VASP with IBRION = 8. Supercells generated by the PHONOPY package were used in the DFPT calculations, and the resulting force constants were subsequently processed with PHONOPY to obtain the phonon dispersion relations and evaluate the dynamical stability [24,25]. Electron–phonon coupling (EPC) calculations were further carried out using Quantum ESPRESSO version 7.2 [26].

Numerical convergence tests were performed by varying the wavefunction cutoff energy from 30 to 80 Ry, the electronic k-point mesh from 16×16×16 to 28×28×28, and the phonon q-point mesh from 4×4×4 to 7×7×7. Based on the convergence of the total energy and the stabilization of both the electron–phonon coupling constant λ and the superconducting transition temperature $T_{c}$ with q-point mesh refinement, a wavefunction cutoff energy of 60 Ry, a 24×24×24 Monkhorst–Pack k-point mesh, and a 6×6×6 q-point mesh were adopted for all production EPC calculations. Scalar-relativistic PBE ultrasoft pseudopotentials from PSLibrary 1.0.0 were employed for the main EPC calculations. To assess the sensitivity of the results to the choice of pseudopotentials, an additional calculation for ${\mathrm{VH}}_{3}$ was performed using SG15 ONCV-PBE v1.2 norm-conserving pseudopotentials for V and H with an 80 Ry wavefunction cutoff and the same 6×6×6 q-point mesh. The superconducting transition temperature $T_{c}$ was estimated within the Allen–Dynes modified McMillan approximation [27], which provides a commonly used comparative estimate based on the EPC constant λ and logarithmic average phonon frequency $\omega_{\log}$.

## 3. Results and Discussion

**Figure 1:**
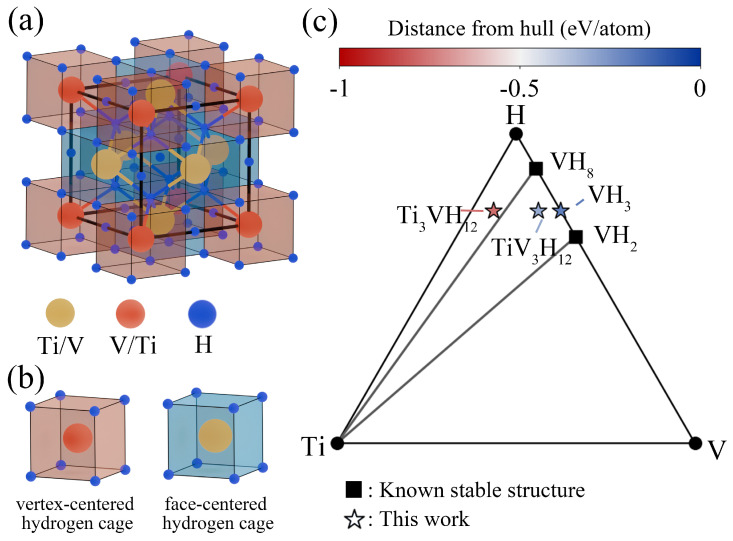
(**a**) Crystal structure of Ti–V–H. (**b**) The vertex-centered hydrogen cage and face-centered hydrogen cage. (**c**) Ternary convex hull at 200 GPa. Black lines are ridges connecting thermodynamically stable points. Star symbols indicate the structure of this work.

${\mathrm{Ti}}_{3}{\mathrm{VH}}_{12}$ and ${\mathrm{TiV}}_{3}H_{12}$ ternary structures were constructed by substituting V atoms at the face-centered and vertex sites of the ${\mathrm{VH}}_{3}$ lattice with Ti atoms, respectively. The resulting structures preserve the three-dimensional hydrogen framework of ${\mathrm{VH}}_{3}$, in which the transition-metal atoms are located at the centers of polyhedral hydrogen cages. Depending on the metal-site occupancy, two distinct local hydrogen environments are formed: a vertex-centered hydrogen cage and a face-centered hydrogen cage. Both cages exhibit cubic configurations. The ball-and-stick model of the structure is shown in Figure 1a, while the local geometries of the vertex and face-centered hydrogen cages are illustrated in Figure 1b. The space groups, lattice parameters, and Wyckoff positions of these structures are listed in Table 1.

After establishing the structural features, the thermodynamic stability of the systems was further evaluated. The thermodynamic stability of the structures was initially evaluated by calculating the formation energy, which is defined as follows:

(1) $$E_{f}=\frac{E\left({\mathrm{Ti}}_{x}V_{y}H_{z}\right)-xE\left(\mathrm{Ti}\right)-yE\left(V\right)-zE\left(H\right)}{x+y+z}.$$

Here, $E_{f}$ denotes the formation energy, $E({\mathrm{Ti}}_{x}V_{y}H_{z})$ is the total energy of the compound at 200 GPa, and E(Ti), E(V), and E(H) represent the average energies of Ti, V, and H atoms at 200 GPa, respectively. The formation energies at 200 GPa were calculated to be −0.91 eV/atom for ${\mathrm{Ti}}_{3}{\mathrm{VH}}_{12}$ and −0.42 eV/atom for ${\mathrm{TiV}}_{3}H_{12}$. For comparison, the formation energy of the prototype ${\mathrm{VH}}_{3}$ structure was also calculated using the same method, yielding a value of −0.24 eV/atom. The lower formation energies of the Ti-substituted structures indicate that both compounds are thermodynamically stable and exhibit higher stability than the original ${\mathrm{VH}}_{3}$ structure.

To assess thermodynamic stability, the formation energies of ${\mathrm{VH}}_{8}$ and ${\mathrm{VH}}_{2}$ at 200 GPa were calculated [28], and the Ti–V–H ternary convex hull was constructed (Figure 1c). ${\mathrm{VH}}_{3}$, ${\mathrm{Ti}}_{3}{\mathrm{VH}}_{12}$, and ${\mathrm{TiV}}_{3}H_{12}$ all lie below the hull, indicating stability at 200 GPa. ${\mathrm{Ti}}_{3}{\mathrm{VH}}_{12}$ has the largest distance from the hull, showing that Ti incorporation lowers system energy and enhances structural stability. These results suggest that transition-metal substitution is effective for stabilizing new Ti–V–H hydrides.

**Figure 2:**
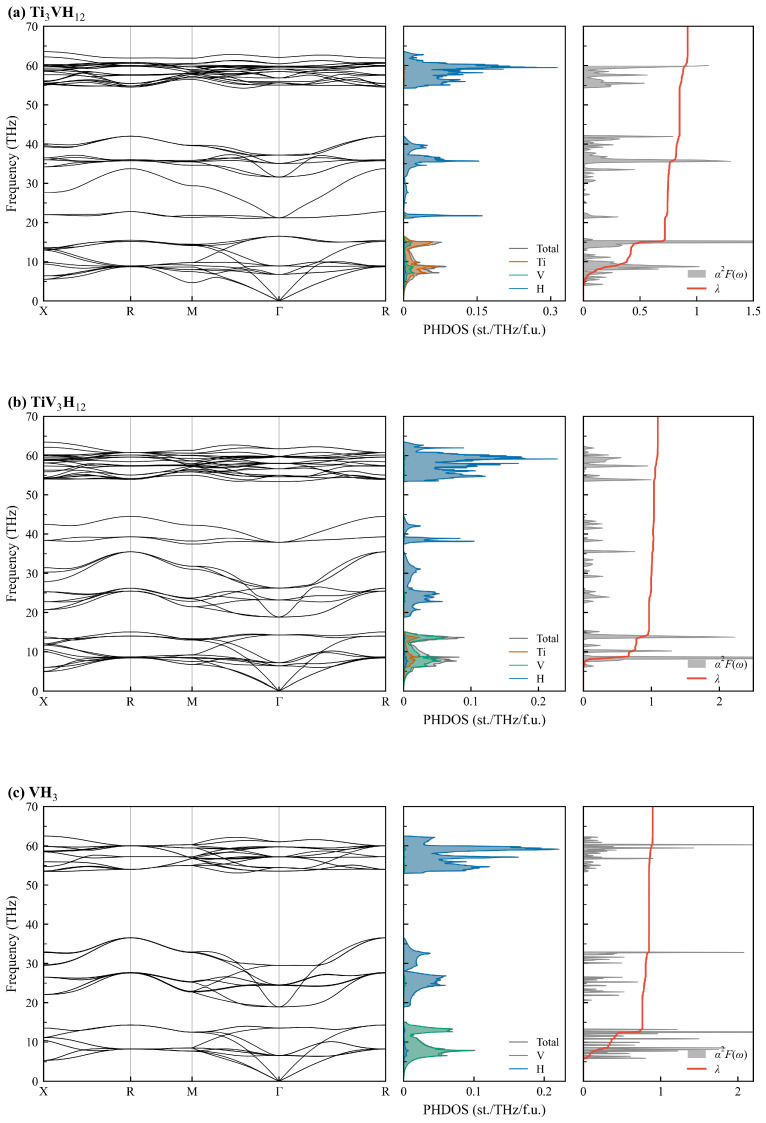
Calculated phonon dispersion curves, phonon density of states, Eliashberg phonon spectral function $\alpha^{2}F\left(\omega \right)$, EPC parameter λ, and the contribution of the phonon modes to the EPC for different frequency ranges of the (**a**) ${\mathrm{Ti}}_{3}{\mathrm{VH}}_{12}$, (**b**) ${\mathrm{TiV}}_{3}H_{12}$, and (**c**) ${\mathrm{VH}}_{3}$.

Phonon dispersion calculations were subsequently performed at 200 GPa to examine the structural stability. Figure 2 presents the phonon dispersion curves of the two newly proposed structures and the prototype ${\mathrm{VH}}_{3}$. No imaginary frequencies are observed throughout the Brillouin zone, indicating that all three structures are dynamically stable at 200 GPa [29]. At this stage, ${\mathrm{Ti}}_{3}{\mathrm{VH}}_{12}$ and ${\mathrm{TiV}}_{3}H_{12}$ can be identified as stable structures. The superconducting properties of both structures were then predicted. For validation, the superconducting properties of ${\mathrm{VH}}_{3}$ were also calculated using the same method. The calculated EPC constant λ, logarithmic average phonon frequency $\omega_{\log}$, density of states at the Fermi level $N(E_{F})$, and superconducting transition temperature $T_{c}$ are summarized in Table 2.

Within the Allen–Dynes modified McMillan approximation, $T_{c}$ was estimated using the following expression:

(2) $$T_{c}=\frac{\omega_{\log}}{1.20}\exp\left[-\frac{1.04(1+\lambda )}{\lambda \left(1-0.62\mu^{*}\right)-\mu^{*}}\right].$$

Here, $\omega_{\log}$ denotes the logarithmic average phonon frequency, λ represents the EPC constant, and $\mu^{*}$ corresponds to the Coulomb pseudopotential. The EPC parameter λ is determined by integrating the Eliashberg spectral function $\alpha^{2}F\left(\omega \right)$ over the entire phonon frequency range, as described by the following equation:

(3) $$\lambda =2\int \frac{\alpha^{2}F\left(\omega \right)}{\omega }\;d\omega .$$

The Eliashberg spectral function $\alpha^{2}F\left(\omega \right)$ and the corresponding λ for the three superconductors exhibiting the $T_{c}$ are presented in Figure 2. In these calculations, the Coulomb pseudopotential $\mu^{*}$ was fixed at 0.1. Although the Eliashberg spectral function contains the full phonon-frequency distribution of the EPC, the Allen–Dynes formula reduces this information to the effective parameters λ and $\omega_{\log}$. Therefore, the $T_{c}$ values reported here should be interpreted as comparative theoretical estimates within the Allen–Dynes approximation rather than as full Migdal–Eliashberg transition temperatures. A full isotropic or anisotropic Migdal–Eliashberg treatment using dense-grid electron–phonon matrix elements, for example through Wannier-interpolated EPW calculations, would be valuable for future quantitative refinement.

The Coulomb pseudopotential $\mu^{*}$ was set to 0.10 as a conventional baseline value widely used for comparative Allen–Dynes estimates of phonon-mediated superconductors. To evaluate the sensitivity of the calculated $T_{c}$ to this parameter, $T_{c}$ was additionally recalculated using $\mu^{*}$ = 0.13. As shown in Table 2, increasing $\mu^{*}$ from 0.10 to 0.13 lowers the absolute $T_{c}$ values from 42.1 to 35.6 K for ${\mathrm{Ti}}_{3}{\mathrm{VH}}_{12}$, from 36.8 to 31.1 K for ${\mathrm{TiV}}_{3}H_{12}$, and from 28.7 to 23.6 K for ${\mathrm{VH}}_{3}$. Nevertheless, the relative trend ${\mathrm{Ti}}_{3}{\mathrm{VH}}_{12}$ > ${\mathrm{TiV}}_{3}H_{12}$ > ${\mathrm{VH}}_{3}$ is maintained, indicating that the main conclusion is not dependent on a single choice of $\mu^{*}$.

The discrepancy between the calculated ${\mathrm{VH}}_{3}$ values and those reported in Ref. [19] was examined explicitly. Using the conventional Coulomb pseudopotential of $\mu^{*}$ = 0.10 in the Allen–Dynes equation, Ref. [19] reports λ = 0.40, $\omega_{\log}$ = 616.0 K, and $T_{c}$ = 2.5 K at 200 GPa, whereas the present ultrasoft-pseudopotential calculation gives λ = 0.84, $\omega_{\log}$ = 558.0 K, and $T_{c}$ = 28.7 K. To assess pseudopotential sensitivity, an additional SG15 ONCV-PBE calculation was performed with an 80 Ry cutoff and a 6×6×6 q-point grid. This calculation gives λ = 0.97, $\omega_{\log}$ = 493.8 K, and $T_{c}$ = 32.9 K. The norm-conserving calculation therefore does not reproduce the lower literature value, indicating that the discrepancy cannot be attributed to the pseudopotential class alone.

Ref. [19] does not specify the exact norm-conserving pseudopotential files, valence configurations, electronic k-point mesh, smearing parameters, or EPC integration details required for a strict one-to-one reproduction. Potential differences in these settings may contribute to the observed variation. The absolute λ and $T_{c}$ values are therefore interpreted as method-dependent Allen–Dynes estimates. Within the internally consistent ultrasoft-pseudopotential framework of this work, ${\mathrm{Ti}}_{3}{\mathrm{VH}}_{12}$ and ${\mathrm{TiV}}_{3}H_{12}$ show higher calculated $T_{c}$ values than ${\mathrm{VH}}_{3}$; this relative trend is discussed with the stated methodological limitation.

From the electron–phonon coupling parameters, the introduction of Ti is found to enhance the electron–phonon interaction in the system, as the EPC constants of ${\mathrm{TiV}}_{3}H_{12}$ (0.99) and ${\mathrm{Ti}}_{3}{\mathrm{VH}}_{12}$ (0.92) are both higher than that of pristine ${\mathrm{VH}}_{3}$ (0.84). However, different effects are observed for $\omega_{\log}$ and the $N(E_{F})$. Both ${\mathrm{Ti}}_{3}{\mathrm{VH}}_{12}$ and ${\mathrm{TiV}}_{3}H_{12}$ exhibit significantly enhanced densities of states at the Fermi level (22.1 and $21.7\;{\mathrm{eV}}^{-1}$, respectively), which favors stronger electron–phonon coupling. Notably, ${\mathrm{Ti}}_{3}{\mathrm{VH}}_{12}$ possesses a much higher $\omega_{\log}$ (683.9 K) than ${\mathrm{TiV}}_{3}H_{12}$ (536.3 K). Consequently, despite the slightly larger EPC constant in ${\mathrm{TiV}}_{3}H_{12}$, the higher phonon characteristic frequency in ${\mathrm{Ti}}_{3}{\mathrm{VH}}_{12}$ leads to a larger superconducting transition temperature. Overall, the variation in $T_{c}$ is governed by the combined effects of λ, $\omega_{\log}$ and $N(E_{F})$, among which ${\mathrm{Ti}}_{3}{\mathrm{VH}}_{12}$ achieves a more favorable balance and therefore exhibits the highest $T_{c}$.

To further analyze the superconducting properties of the system, the phonon dispersions, phonon density of states (PHDOS), Eliashberg spectral function $\alpha^{2}F\left(\omega \right)$, and the cumulative electron–phonon coupling parameter λ(ω) for ${\mathrm{Ti}}_{3}{\mathrm{VH}}_{12}$, ${\mathrm{TiV}}_{3}H_{12}$, and ${\mathrm{VH}}_{3}$ are presented in Figure 2. The phonon spectra of the three structures can be roughly divided into three regions: the low-frequency region (<20 THz), the intermediate-frequency region (20–50 THz), and the high-frequency region (>50 THz). The low-frequency modes are mainly associated with vibrations of the transition-metal atoms, whereas the intermediate- and high-frequency modes are dominated by H vibrations. The contributions to the EPC are found to be approximately 78%, 88%, and 84% from the low-frequency region, 14%, 7%, and 11% from the intermediate-frequency region, and 8%, 5%, and 5% from the high-frequency region for ${\mathrm{Ti}}_{3}{\mathrm{VH}}_{12}$, ${\mathrm{TiV}}_{3}H_{12}$, and ${\mathrm{VH}}_{3}$, respectively.

These results indicate that the EPC in all three structures is mainly dominated by low-frequency vibrations of transition-metal atoms, while H-derived intermediate- and high-frequency modes contribute less directly. This behavior is consistent with the definition of the EPC constant (Equation (3)) in which the 1/ω weighting amplifies the contribution of low-frequency phonons to the total EPC strength. Therefore, the metal-dominated low-frequency modes are primarily responsible for the large λ values, whereas the H-dominated intermediate- and high-frequency modes help maintain relatively large $\omega_{\log}$ values. The calculated $T_{c}$ is thus controlled by the combined effect of low-frequency metal vibrations and high-frequency H vibrations rather than by the H-derived modes alone. Nevertheless, owing to their high frequencies, H vibrations play a key role in determining $\omega_{\log}$ and thus indirectly enhance $T_{c}$. Compared with other hydrogen-cage superconductors, such as the octahedral hydrogen-cage structures of ${\mathrm{Mg}}_{2}{\mathrm{IrH}}_{6}$ [30] and ${\mathrm{Li}}_{2}{\mathrm{AuH}}_{6}$ [31], as well as the cubic hydrogen-cage structure of ${\mathrm{LaBeH}}_{8}$ [32], the hydrogen contribution to EPC in the present system is significantly reduced, suggesting that the ${\mathrm{VH}}_{3}$-type cage partially suppresses H-derived coupling. Furthermore, Ti substitution markedly increases the density of states at the Fermi level and strengthens EPC, leading to higher $T_{c}$ in ${\mathrm{Ti}}_{3}{\mathrm{VH}}_{12}$ and ${\mathrm{TiV}}_{3}H_{12}$ than in ${\mathrm{VH}}_{3}$. The underlying electronic structures are further analyzed below.

**Figure 3:**
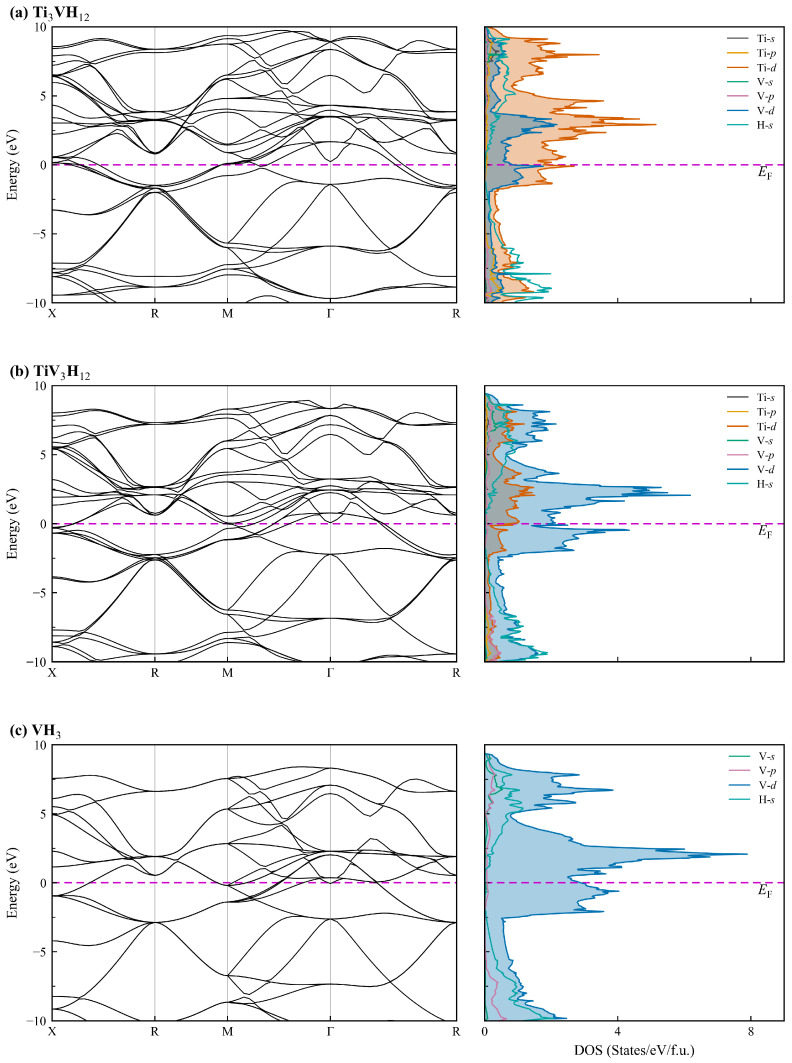
Calculated band structures and the DOS of (**a**) ${\mathrm{Ti}}_{3}{\mathrm{VH}}_{12}$, (**b**) ${\mathrm{TiV}}_{3}H_{12}$, and (**c**) ${\mathrm{VH}}_{3}$.

The electronic band structures and projected density of states (PDOS) of ${\mathrm{Ti}}_{3}{\mathrm{VH}}_{12}$, ${\mathrm{TiV}}_{3}H_{12}$, and ${\mathrm{VH}}_{3}$ are presented in Figure 3. It can be observed that multiple bands cross the Fermi level in all three structures, indicating pronounced metallic behavior. From the PDOS analysis, the electronic states near the Fermi level are found to be primarily contributed by the *d* orbitals of the transition metals, accompanied by metal–hydrogen orbital hybridization. Notably, a pronounced peak in the density of states can be observed near the Fermi level in ${\mathrm{Ti}}_{3}{\mathrm{VH}}_{12}$ and ${\mathrm{TiV}}_{3}H_{12}$, which may be associated with van Hove-related features close to $E_{F}$. These features increase the electronic states available near the Fermi level and can favor stronger electron–phonon coupling, but they should be regarded as one contributing factor rather than the sole origin of the calculated $T_{c}$ enhancement [33].

In contrast, the main DOS peak in ${\mathrm{VH}}_{3}$ is located farther below the Fermi level, so it contributes less directly to the states exactly at $E_{F}$ than the near-$E_{F}$ features in the Ti-containing compounds. However, this electronic feature alone cannot fully explain the difference in $T_{c}$. Accordingly, the differences in $T_{c}$ among these compounds should be interpreted as the combined outcome of $N(E_{F})$, phonon frequencies, electron–phonon matrix elements, λ, and $\omega_{\log}$, rather than as a direct consequence of a single DOS peak.

Further comparison between ${\mathrm{Ti}}_{3}{\mathrm{VH}}_{12}$ and ${\mathrm{TiV}}_{3}H_{12}$ reveals noticeable differences in their electronic structures near $E_{F}$. In ${\mathrm{Ti}}_{3}{\mathrm{VH}}_{12}$, the van Hove singularity is located very close to the Fermi level and appears as a pronounced maximum, whereas in ${\mathrm{TiV}}_{3}H_{12}$ the PDOS shows only a weak peak at $E_{F}$, with the main DOS maximum slightly shifted away from the Fermi level. Therefore, by further tuning the Fermi level—such as through pressure modulation [34,35,36] or additional Ti substitution—the density of states in ${\mathrm{TiV}}_{3}H_{12}$ may be further enhanced, suggesting a potential route for achieving an even higher $T_{c}$.

**Figure 4:**
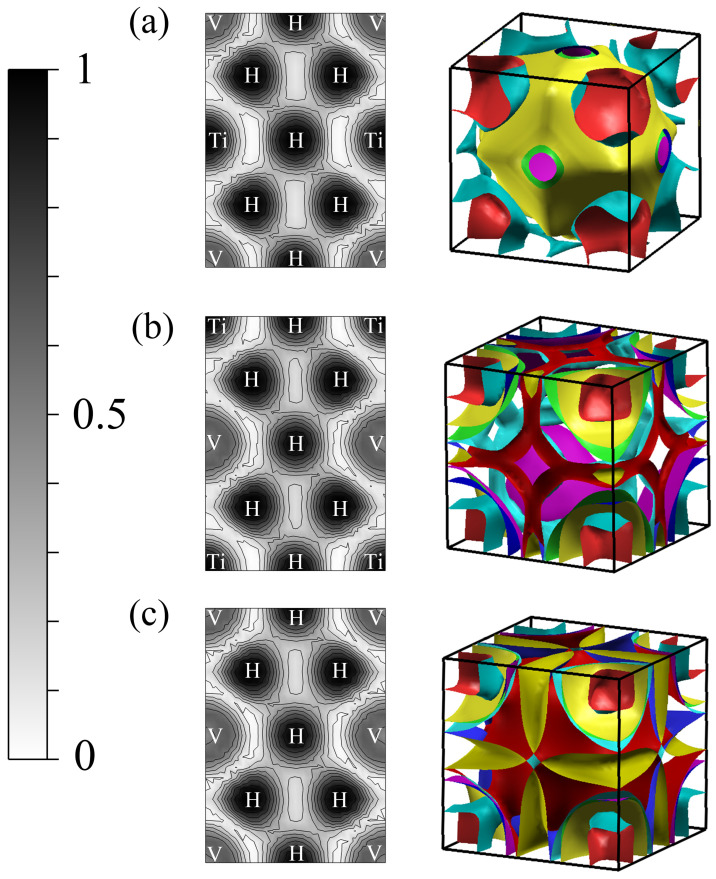
Electronic localization function (ELF) and Fermi surface topology of (**a**) ${\mathrm{Ti}}_{3}{\mathrm{VH}}_{12}$, (**b**) ${\mathrm{TiV}}_{3}H_{12}$, and (**c**) ${\mathrm{VH}}_{3}$.

**Figure 5:**
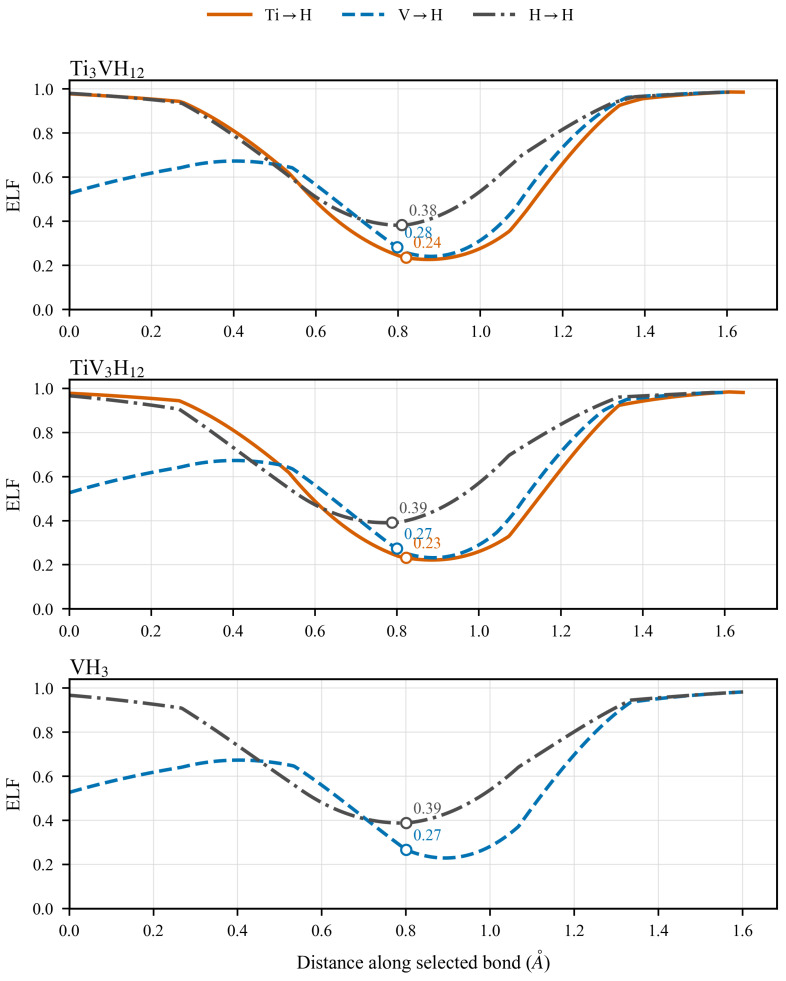
ELF line profiles along selected nearest-neighbor bonds in ${\mathrm{Ti}}_{3}{\mathrm{VH}}_{12}$, ${\mathrm{TiV}}_{3}H_{12}$, and ${\mathrm{VH}}_{3}$. The horizontal axis represents distance from the first atom indicated in the legend toward the second atom; open circles denote the corresponding bond-midpoint ELF values.

The ELF and Fermi surface topology were calculated to elucidate the electronic characteristics and metal–hydrogen interactions (Figure 4). The ELF was taken along the (110) plane, clearly revealing the electronic distribution between transition-metal and hydrogen atoms. In all structures, electrons are mainly localized around H atoms, while more delocalized behavior is observed between transition-metal atoms. Finite electron density between metal and hydrogen atoms indicates the presence of metal–hydrogen hybridization. To resolve the bonding-related ELF distribution quantitatively, ELF line profiles were extracted along representative nearest-neighbor Ti–H, V–H, and H–H bonds (Figure 5). The horizontal coordinate represents the distance from the first atom indicated in the legend toward the second atom (Ti→H, V→H, or H→H), and the open circles mark the bond midpoints. The midpoint ELF values are 0.236, 0.282, and 0.383 for Ti–H, V–H, and H–H in ${\mathrm{Ti}}_{3}{\mathrm{VH}}_{12}$; 0.231, 0.274, and 0.391 in ${\mathrm{TiV}}_{3}H_{12}$; and 0.266 and 0.387 for V–H and H–H in ${\mathrm{VH}}_{3}$, respectively. These results indicate that the V–H bond region exhibits a higher ELF than the Ti–H bond region, whereas the H–H bond midpoint shows the strongest electron localization. The ELF analysis therefore serves to characterize the degree of electron localization along representative chemical bonds. However, the enhancement of $T_{c}$ cannot be attributed to ELF alone; instead, it arises from the combined effects of the electronic states near $E_{F}$, phonon spectra, and electron–phonon coupling (EPC).

The Fermi surface topology further reveals the distribution of electronic states near the Fermi level. All three structures exhibit multiple three-dimensional Fermi surface sheets, indicating multi-band contributions to conduction. With increasing Ti content, the Fermi surface becomes more complex, featuring more sheets and enhanced three-dimensional connectivity. This complexity suggests additional electron scattering channels that favor stronger electron–phonon interaction. Combined with the density of states and van Hove-related feature analysis, these results indicate that Ti substitution modifies both the electronic structure and Fermi surface topology, enabling more electronic states to participate in electron–phonon scattering which may provide additional electronic channels for EPC. The resulting $T_{c}$ enhancement should therefore be attributed to the coupled effects of electronic states at $E_{F}$, phonon spectra, and EPC strength.

**Figure 6:**
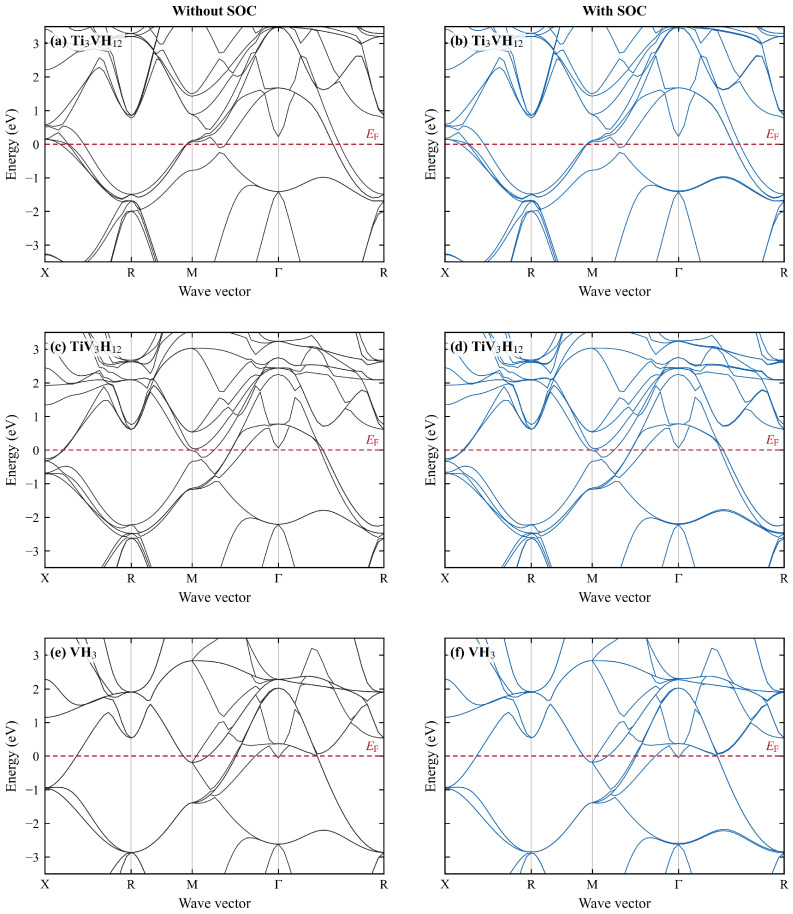
Comparison of the calculated electronic band structures without and with spin–orbit coupling (SOC) for (**a**,**b**) ${\mathrm{Ti}}_{3}{\mathrm{VH}}_{12}$, (**c**,**d**) ${\mathrm{TiV}}_{3}H_{12}$, and (**e**,**f**) ${\mathrm{VH}}_{3}$. The Fermi level ($E_{F}$) is set to zero.

**Figure 7:**
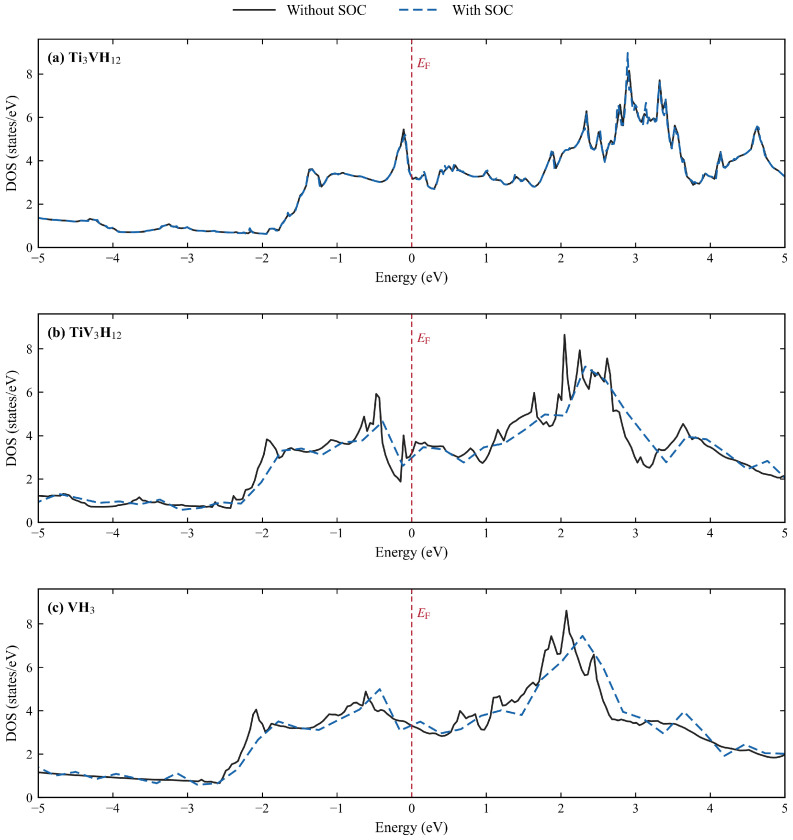
Comparison of the calculated total density of states (DOS) without and with spin–orbit coupling (SOC) for (**a**) ${\mathrm{Ti}}_{3}{\mathrm{VH}}_{12}$, (**b**) ${\mathrm{TiV}}_{3}H_{12}$, and (**c**) ${\mathrm{VH}}_{3}$. The Fermi level ($E_{F}$) is set to zero.

To evaluate the effect of spin–orbit coupling (SOC), additional SOC calculations were performed. The electronic band structures and density of states with and without SOC (Figure 6 and Figure 7) show only negligible differences, indicating that SOC has little influence on the electronic structure near the Fermi level. Therefore, SOC was neglected in the subsequent electron–phonon coupling calculations.

## 4. Conclusions

In summary, the structural stability and superconducting properties of ${\mathrm{Ti}}_{3}{\mathrm{VH}}_{12}$ and ${\mathrm{TiV}}_{3}H_{12}$ were investigated using first–principles calculations at 200 GPa. Both compounds exhibit negative formation energies and no imaginary phonon modes, and they are located below the Ti–V–H convex hull under the studied pressure condition, indicating thermodynamic and dynamical stability. Calculations of the superconducting properties show that the electron–phonon coupling in these systems is mainly associated with low-frequency transition-metal vibrations, whereas high-frequency hydrogen vibrations contribute to the logarithmic average phonon frequency. Within the common ultrasoft-pseudopotential calculation framework, ${\mathrm{Ti}}_{3}{\mathrm{VH}}_{12}$ and ${\mathrm{TiV}}_{3}H_{12}$ display enhanced densities of states near the Fermi level and higher calculated $T_{c}$ values than ${\mathrm{VH}}_{3}$. The ${\mathrm{VH}}_{3}$ cross-check indicates that absolute λ and $T_{c}$ values remain sensitive to methodological choices and should be interpreted as comparative theoretical estimates. The electronic-structure analysis suggests that Ti substitution tunes *d*-derived states and places van Hove singularities close to the Fermi level, while ELF and Fermi-surface results indicate modified metal–hydrogen hybridization and more complex conducting sheets. These findings support the use of Ti–V–H hydrides as a model system for understanding how transition-metal substitution can link framework stability, Fermi-level tuning, and electron–phonon coupling in compressed hydride superconductors.

## Figures and Tables

**Table 1:** Space group, lattice parameters, and Wyckoff positions of ${\mathrm{Ti}}_{3}{\mathrm{VH}}_{12}$, ${\mathrm{TiV}}_{3}H_{12}$, and ${\mathrm{VH}}_{3}$.

Structure	Space Group	Lattice Parameters	Wyckoff Positions		
			Ti	V	H
${\mathrm{Ti}}_{3}{\mathrm{VH}}_{12}$	$Pm\bar{3}m$	a=3.76 Å	3c (0.0, 0.5, 0.5)	1a (0.0, 0.0, 0.0)	1b (0.5, 0.5, 0.5); 3d (0.0, 0.0, 0.5); 8g (0.25, 0.25, 0.25)
${\mathrm{TiV}}_{3}H_{12}$	$Pm\bar{3}m$	a=3.72 Å	1a (0.0, 0.0, 0.0)	3c (0.0, 0.5, 0.5)	1b (0.5, 0.5, 0.5); 3d (0.0, 0.0, 0.5); 8g (0.25, 0.25, 0.25)
${\mathrm{VH}}_{3}$	$Fm\bar{3}m$	a=3.70 Å	—	4a (0.0, 0.5, 0.5)	4b (0.0, 0.0, 0.0); 8c (0.25, 0.25, 0.25)

**Table 2:** Electron–phonon coupling constant (λ), logarithmic average phonon frequency ($\omega_{\log}$, K), density of states at the Fermi level ($N(E_{F})$, ${\mathrm{eV}}^{-1}$), and superconducting transition temperature ($T_{c}$, K) of ${\mathrm{Ti}}_{3}{\mathrm{VH}}_{12}$, ${\mathrm{TiV}}_{3}H_{12}$, and ${\mathrm{VH}}_{3}$. Ref. [19] and the norm-conserving pseudopotential (nc) calculations are compared for ${\mathrm{VH}}_{3}$.

Structure	$\mu^{*}$	λ	$\omega_{\log}$	$N(E_{F})$	$T_{c}$
${\mathrm{Ti}}_{3}{\mathrm{VH}}_{12}$	0.10	0.92	683.9	22.1	42.1
${\mathrm{Ti}}_{3}{\mathrm{VH}}_{12}$	0.13	0.92	682.3	22.1	35.6
${\mathrm{TiV}}_{3}H_{12}$	0.10	0.99	536.3	21.7	36.8
${\mathrm{TiV}}_{3}H_{12}$	0.13	0.97	544.4	22.9	31.1
${\mathrm{VH}}_{3}$	0.10	0.84	558.0	12.9	28.7
${\mathrm{VH}}_{3}$	0.13	0.88	545.9	20.6	23.6
${\mathrm{VH}}_{3}$ [19]	—	0.40	616.0	51.7	2.5
${\mathrm{VH}}_{3}$-nc	0.10	0.97	493.8	22.2	32.9
${\mathrm{VH}}_{3}$-nc	0.13	0.97	493.8	22.2	28.0

## Data Availability

The raw data supporting the conclusions of this article will be made available by the authors upon request.

## Abbreviations

The following abbreviations are used in this manuscript:

DFT	Density functional theory
DFPT	Density functional perturbation theory
DOS	Density of states
EPC	Electron–phonon coupling
ELF	Electron localization function
PDOS	Projected density of states
PHDOS	Phonon density of states
SOC	Spin–orbit coupling
